# Whole genome sequencing of Malaysian colorectal cancer patients reveals specific druggable somatic mutations

**DOI:** 10.3389/fmolb.2022.997747

**Published:** 2023-02-14

**Authors:** Ryia Illani Mohd Yunos, Nurul-Syakima Ab Mutalib, Jia-shiun Khoo, Sazuita Saidin, Muhiddin Ishak, Saiful Effendi Syafruddin, Francis Yew Fu Tieng, Najwa Farhah Md Yusof, Mohd Ridhwan Abd Razak, Norshahidah Mahamad Nadzir, Nadiah Abu, Isa Md Rose, Ismail Sagap, Luqman Mazlan, Rahman Jamal

**Affiliations:** ^1^ UKM Medical Molecular Biology Institute (UMBI), Kuala Lumpur, Malaysia; ^2^ Codon Genomics SB, Seri Kembangan, Selangor, Malaysia; ^3^ Department of Pathology, Faculty of Medicine, Universiti Kebangsaan Malaysia, Kuala Lumpur, Malaysia; ^4^ Department of Surgery, Faculty of Medicine, Universiti Kebangsaan Malaysia, Kuala Lumpur, Malaysia

**Keywords:** Malaysians colorectal cancer, whole genome sequencing, precision medicine, druggable alterations, RNF43, KDM4E, MUC16, POTED

## Abstract

The incidences of colorectal cancer (CRC) are continuously increasing in some areas of the world, including Malaysia. In this study, we aimed to characterize the landscape of somatic mutations using the whole-genome sequencing approach and identify druggable somatic mutations specific to Malaysian patients. Whole-genome sequencing was performed on the genomic DNA obtained from 50 Malaysian CRC patients’ tissues. We discovered the top significantly mutated genes were APC, TP53, KRAS, TCF7L2 and ACVR2A. Four novel, non-synonymous variants were identified in three genes, which were KDM4E, MUC16 and POTED. At least one druggable somatic alteration was identified in 88% of our patients. Among them were two frameshift mutations in *RNF43* (G156fs and P192fs) predicted to have responsive effects against the Wnt pathway inhibitor. We found that the exogenous expression of this RNF43 mutation in CRC cells resulted in increased cell proliferation and sensitivity against LGK974 drug treatment and G1 cell cycle arrest. In conclusion, this study uncovered our local CRC patients’ genomic landscape and druggable alterations. It also highlighted the role of specific *RNF43* frameshift mutations, which unveil the potential of an alternative treatment targeting the Wnt/β-Catenin signalling pathway and could be beneficial, especially to Malaysian CRC patients.

## 1 Introduction

Colorectal cancer (CRC) is among the top three most common cancer worldwide, with 1.93 million new cases and 900,00 deaths reported in 2020 (Sung et al., 2021; Xi and Xu, 2021). Asian countries have also experienced a significant hike in CRC incidences in these past 20 years, especially with the changes in lifestyle and diet (Arnold et al., 2017). In Malaysia, CRC is the most common cancer among men and the second most common among women (Hashimah et al., 2019). According to the National *Cancer* Patient Registry-Colorectal *Cancer*, from 2008 to 2013, 4,501 cases of CRC were reported, most of which were Chinese, followed by Malays and Indians (Abu Hassan et al., 2016).

Substantial efforts have been made to understand the basic molecular mechanisms of CRC through profiling of somatic mutations, including the International *Cancer* Genome Consortium (Hudson et al., 2010), The *Cancer* Genome Atlas (TCGA) (The Cancer Genome Atlas Network, 2012) and the Pan-Cancer Analysis of Whole Genomes (PCAWG) (Campbell et al., 2020). However, there is still a lack of understanding in using the publicly available information to treat CRC patients effectively and unfortunately, their potential clinical significances are largely unexplored. Prognostication and treatment decision-making have been improved by numerous biomarkers discovered through comprehensive molecular profiling (Sveen et al., 2020). Several biomarkers and prognostic values, such as *KRAS* and *EGFR*, have been widely studied (Grady and Pritchard, 2014). Despite many studies on *KRAS* as a biomarker, the Ras protein has not yielded any therapeutic intervention due to the absence of a suitable site to which drugs could bind (McCormick, 2015; Liu et al., 2019). Alternatively, studies have been focused on blocking the pathways downstream of RAS, especially the RAF-MAPK pathway and the PI3 kinase pathways, to provide clinical benefit for patients with Ras-associated cancer (McCormick, 2015). Thus, by exploring the landscape of the alterations in cancer patients, new possible therapeutic targets and clinically relevant somatic mutations may be identified.

One of the most important signalling pathways implicated in CRC pathogenesis is the Wnt/β-catenin signalling pathway (Cheng et al., 2019) which is involves in various physiological and developmental processes such as proliferation, differentiation, apoptosis, migration, invasion and tissue homeostasis (Clevers and Nusse, 2012; Ng et al., 2019). Dysregulation of the pathway may contribute to the development and progression of specific solid tumours and haematological malignancies. (Cheng et al., 2019; Zhang and Wang, 2020). There has been increasing evidence supporting the potential relevance of the Wnt/β-catenin signalling pathway as a therapeutic target in cancer treatment (Blagodatski et al., 2014; Zhang and Wang, 2020). One of the components of this pathway is *RNF43* (E3 ubiquitin-protein ligase RNF43), a type of ubiquitin ligase located in the transmembrane region (Zebisch and Jones, 2015). In cancer cells, Wnt signalling is activated through loss of function of *RNF43 via* mutations, leading to a decrease in the degradation of Frizzled (Serra and Chetty, 2018). Studies have shown that *RNF43* mutations can have dual roles, either as a negative or positive regulator of the Wnt/β-catenin signalling pathway, depending on the type and location of the mutations in the gene (Yu et al., 2020; Cho et al., 2022; Fang et al., 2022). Somatic mutations in *RNF43* have been associated with increased sensitivity to compounds that target the Wnt pathway, such as the porcupine (PORCN) inhibitor LGK974. LGK974 impairs the PORCN protein that will subsequently suppress the post-translational acylation of Wnt-ligands and inhibit their secretion. Consequently, it prevents the activation of Wnt ligands, dysregulates the Wnt-mediated signalling, and inhibits cell growth in Wnt-driven tumours (Liu et al., 2013). Therefore, as the PORCN inhibitors and other upstream inhibitors advance into clinical trials, it is essential to identify the suitable patients to be treated with these Wnt inhibitors. Hence, a comprehensive map of druggable mutations is required.

Whole-genome sequencing (WGS) can provide insight into the mutational spectra of cancers across the entire genome. In the past decades, several new promising therapeutic targets have been discovered through this approach. Extensive reviews and studies on how germline and somatically derived variants can guide therapeutic decisions have been carried out, which highlighted the importance of genome profiling of cancer (Jia et al., 2014; Ab Mutalib et al., 2017; Chan et al., 2019; Yang H. et al., 2019). Moreover, personal genome sequencing may become essential for diagnosing, preventing, and treating human diseases, particularly cancer (Cragun et al., 2016). Patient care can also be improved by transforming genomic research into personalized medicine applications by developing new and better genomics-based diagnostic tests. In this study, we employed WGS to characterize the landscape of somatic alterations in 50 Malaysian CRC patients, identify somatic alterations suitable for anticancer drug treatment, and predict the drug response. In addition, we functionally characterized two novel *RNF43* variants and demonstrated that these are potentially clinically relevant variants worth exploring in future studies.

## 2 Materials and methods

### 2.1 Clinical materials

A total of 50 Malaysian CRC patients were enrolled from 2010 to 2018. All the individuals gave their written informed consent, and the study was approved under UKM PPI/111/8/JEP-2017–583. All the patients were categorized according to clinicopathological characteristics such as the age of diagnosis, ethnicity, gender, TNM classification, metastasis status, differentiation, tumour localization and survival status. Fifty paired colorectal carcinoma and their corresponding blood DNA or adjacent normal tissues were collected. The collected tissues were subjected to H&E staining and only tissues with 80% of tumour cells, confirmed by the pathologist, were selected to be used in the present study. DNA extraction was performed using AllPrep DNA/RNA/miRNA universal Kit (Qiagen, Germany) according to the manufacturer’s protocol. The quantity of the extracted DNA was assessed using Qubit Fluorometer (Thermo Fisher Scientific, United States). The quality of the extracted DNA was evaluated by agarose gel electrophoresis and NanoDrop 2000 spectrophotometer (Thermo Fisher Scientific, United States). To confirm the identity and to avoid contamination of each tumour and blood or normal tissue paired samples, we profiled the DNA based on 15 polymorphic STR markers using Investigator^®^ IDplex Plus (Qiagen, Germany). The microsatellite status of each patient was determined using MSI Analysis System, Version 1.2 (Promega Corporation, United States) according to the manufacturer’s protocol. The amplified fragments were detected on the 3130xl Genetic Analyzer (Applied Biosystem, United States).

### 2.2 Library construction and Whole Genome sequencing

Libraries for WGS were constructed using TruSeq^®^ Nano DNA HT Library Prep Kit (Illumina, United States) according to the manufacturer’s protocol. One μg (1 μg) of genomic DNA was randomly fragmented by the S220 Covaris instrument (Covaris, country, United Kingdom) following the manufacturer’s protocol. The fragmented DNA was viewed using gel electrophoresis and purified using AxyPrep Mag PCR clean-up kit (Thermo Fisher Scientific, United States) and then underwent end-repairing, phosphorylation and A-tailing reactions. WGS was performed as 150 bp paired-end, with the average coverage of at least 30X, on Illumina HiSeq X-Ten (Illumina, United States).

### 2.3 Bioinformatic analysis

We utilized FASTQC v0.10.01 software to perform adapter trimming and removal of low quality (less than Q30), short and ambiguous reads (Andrews, 2010). The resulting clean reads were then aligned to the reference human genome (UCSC hg19; http://genome.ucsc.edu/) (International Human Genome Consortium et al., 2001) using the Burrows-Wheeler Aligner (BWA) MEM (Li and Durbin, 2010). Picard tools and Genome Analysis Tool Kit (GATK) IndelRealigner and BaseRecalibrator were adopted to remove duplicate reads, base quality score recalibration and indel realignment. Somatic single nucleotide variants (SNVs) and insertion deletions (Indels) calling were carried out for each pair of tumour-normal samples using MuTect2 (Cibulskis et al., 2013). Functional annotation of the variants identified was performed on 1st February 2016 using ANNOVAR (Wang et al., 2010). Annotations, against Ensembl database, for mutation function (including frameshift insertion/deletion, non-frameshift insertion/deletion, synonymous SNV, non-synonymous SNV, stopgain and stoploss), mutation location (including exonic, intronic, splicing, upstream, downstream, 3′untranslated region (UTR) and 5′UTR), amino acid changes, allele frequencies (based on 1000 Genomes Project, Exome Aggregation Consortium (ExAC), Exome Sequencing Project v.6500 (ESP6500) data) and dbSNP (version 144), as well as COSMIC (v70-14th August 2014), were performed. Finally, all detected variants were manually reviewed using the Integrative Genomics Viewer (IGV) (Thorvaldsdóttir et al., 2013). cBioPortal was utilized to visualize the location of the identified mutations in the gene compared to other publicly available datasets (https://www.cbioportal.org/mutation_mapper) (Cerami et al., 2012; Gao et al., 2013).

### 2.4 Single nucleotide variant and indels variants prioritization

Variants with a quality score above Q30 were considered for further analysis. Frequent variants were removed based on a minimal allele frequency (MAF) threshold of more than 5% from 1000 Genomes Project, ExAC and ESP6500 databases. Besides, variants not resulting in amino acid changes and/or identified in unannotated genes (unknown) and non-exonic regions (based on Ensembl) were also removed. Somatic variants were identified by excluding those specified in both tumour and normal samples and considered a true novel if the variant has not been reported in both dbSNP and COSMIC databases. We ensured that the corresponding normal sample has at least ten reads covering the position with zero variant reads for each of the novel somatic mutation candidates identified. For the resulting candidate of somatic mutations, the alignment of each sample was manually examined for possible mapping ambiguities and sequencing artefacts using Intergrative Genomics Viewer (IGV). Finally, we assessed the potential functional effects of each identified somatic variant based on protein impact prediction tools, SIFT and PolyPhen2.

### 2.5 Druggable and tumor driver alterations

We employed *Cancer* Genome Interpreter (Tamborero et al., 2018) to assess the relevance of the shortlisted somatic alterations as biomarkers of drug response and identify possible tumour driver alterations. Colorectal adenocarcinoma (COREAD) was selected as a cancer type for annotation.

### 2.6 Variants validation by sanger sequencing

All the shortlisted SNVs were validated using the Sanger sequencing method on both tumour and matched blood samples. Primers corresponding to the selected locations were designed using PrimerQuest (Integrated DNA Technologies, United States). PCR products were generated and purified using QIAquick PCR Purification Kit (Qiagen, Germany) and cycle sequencing was performed using the BigDye™ Terminator V3.1 reagent (Applied Biosystem, United States). The cycle sequencing products were then processed using ethanol precipitation, and sequencing was carried out using the 3130xl Genetic Analyzer (Applied Biosystem, United States). The results were analyzed using the Sequence Scanner software (Applied Biosystem, United States).

### 2.7 Lentiviral vectors construction

The RNF43 coding sequences harbouring the mutations of interest, G156Afs and p.P192Gfs, were purchased from Origene Technologies (United States). The wild-type RNF43 coding sequence was purchased from Genscript (United States). These wild-type and mutant RNF43 coding sequences were amplified to incorporate the 3x FLAG upstream of the start codon, and the EcoRI and XbaI restriction sites at the 5′ and 3′ ends, respectively, for sub-cloning purpose into the expression vector pLVX-Puro (Clontech Laboratories Inc., United States). The RNF43 coding sequences were ligated into the expression vector using T4 Ligase (New England Biolabs) according to the manufacturer’s recommendation and transformed into the chemically competent DH5α *E.coli*.

### 2.8 Cell lines, lentiviral transduction and transient transfection

HEK293T cells were used for lentivirus production and cultured in DMEM (Nacalai Tesque, Japan), supplemented with 10% (v/v) fetal bovine serum (FBS) (Nacalai Tesque, Japan) and 1% (v/v) penicillin-streptomycin mixed solution (Nacalai Tesque, Japan). HEK293T cells were seeded at 1.2 × 10^6^ cells/well in six wells plate a day before transfection. The RNF43-expressing plasmid was co-transfected into the HEK293T cells along with packaging plasmids psPAX2 (Addgene, United States) and pMD2.G (Addgene, United States) using Lipofectamine 2000 according to the manufacturer’srecommendation. The media containing the lentiviral were collected 72 h post-transfection and filtered through a 0.45 μM PVDF sterile filter (Merck Milipore, Germany). For the lentiviral transduction, the SW48 cells were seeded into the six wells plate at a density of 2.5 × 10^6^ cells/well, and transduced with the collected supernatant on the following day in the presence of 8 μg/ml polybrene (Merck Millipore, Germany).

### 2.9 Gene expression and protein analysis

Total RNA was extracted from the cell line using the AllPrep DNA/RNA mini kit (Qiagen, Germany) according to the manufacturer’s protocol and cDNA was synthesized using the iScript cDNA synthesis kit (Bio-rad Laboratories Inc., United States). Quantitative PCR (qPCR) was performed using Sso Advanced™ universal SYBR^®^ green mastermix (Bio-Rad Laboratories Inc., United States) and run on CFX96 Real-Time PCR Detection System (Bio-Rad Laboratories, United States). GAPDH and *β*-actin were used as standardization controls, and fold change was calculated based on 2−∆∆Ct (Schmittgen and Livak, 2008).

Total protein from cell cultures was extracted using RIPA buffer and resolved on 10% acrylamide gel. The target protein was analyzed against the following primary antibody: Anti Flag M2 (Sigma Aldrich, United States, 1:1,000) and the secondary antibody was Rabbit anti-mouse IgG/HRP conjugated (Dako, Denmark, 1:1,000).

### 2.10 Ki67 proliferation assay

Muse^®^ Ki67 Proliferation kit (Merck Milipore, Germany) was used to determine the percentage of proliferating cells based on Ki67 expression according to the manufacturer’s protocol. The stained cells were analyzed on the Muse^®^ Cell Analyzer (Merck Milipore, Germany), followed by data analysis using Muse 1.7 Analysis software (Merck Milipore, Germany).

### 2.11 LGK974 drug sensitivity assay

The SW48 cells expressing wild type (Flagged-RNF43^WT^) and mutant RNF43 proteins (Flagged-RNF43^p.G156fs^ and Flagged-RNF43^p.P192fs^) were treated with different concentrations of LGK974 ranging from 10 to 100 µM for 48 h. The control groups, cells treated with 0.1 and 1% (v/v) of DMSO, were included in each experiment. The cell viability was analyzed by using the XTT Cell Viability Assay Kit (Biotium, Germany) based on the manufacturer’s user guide to assess the sensitivity of the cells against the drug treatment.

### 2.12 Cell cycle assay

Cell cycle analysis was performed on SW48 cells expressing wild type (Flagged-RNF43^WT^) and mutant RNF43 proteins (Flagged-RNF43^p.G156fs^ and Flagged-RNF43^p.P192fs^) treated with 50 µM LGK974 for 48 h. The harvested cells were stained with propidium iodide provided in BD Cycletest™ Plus DNA Reagent Kit (BD Biosciences, US) according to the manufacturer’s instructions. The DNA content of at least 10,000 cells was analyzed by FACS Aria II flow cytometry (BD Biosciences, United States) for each experiment before the data was analyzed using ModFit LT 5.0 (Verity Software House, United States).

## 3 Results

### 3.1 Patients characteristic

The characteristics of all 50 patients are listed in Table 1. Most patients were in stage 3, 70% (*n* = 35). The average age of patients was approximately 64 years old (range 30 – 89 years old). The samples comprised an equal number of well-differentiated adenocarcinomas and moderately differentiated adenocarcinoma. Of these 50 patients, 36% (*n* = 18) have died and 54% (*n* = 27) are still alive (as when the data were collected in 2018).

**TABLE 1:** Clinicopathological data of 50 colorectal cancer patients.

Characteristics	Number of patients, n (%)
**All patients**	50 (100)
**Age**	
Average	64
>50	46 (92)
<50	4 (8)
**Gender**	
Male	28 (56)
Female	22 (44)
**Race/Ethnicity**	
Malay	37 (74)
Chinese	13 (26)
Indian	0 (0)
**Stage**	
T1	3 (6)
T2	6 (12)
T3	35 (70)
T4	6 (12)
Metastasized to Lymph Nodes	27
Non-Metastasized	23
**Differentiation**	
Moderately Differentiated	25 (50)
Well Differentiated	25 (50)
Poorly Differentiated	0 (0)
**Vital Status**	
Deceased	18 (36)
Alive	27 (54)
Untraceable	5 (10)

### 3.2 Whole Genome sequencing analysis and coverage

A total of 100 (50 pairs) tumour and normal samples were sequenced using the Illumina HiSeq X Ten platform. At least 730 million reads were generated for each sample, producing approximately 30× to 50× sequencing depth. The reference mapping of the data against human genome hg19 and alignment refinement were carried out based on GATK Best Practices. On average, ∼99% of the reads were found to align to hg19, with at least 83% achieving more than 20× coverage (Table 2).

**TABLE 2:** Summary of sequencing statistics in each sample.

Sample ID	Number of Reads	Read Size (Gb)	Uniquely mapped reads (%)	Sequencing coverage (×)	Sequencing coverage ≥10× (%)	Sequencing coverage ≥20× (%)
C187	709,287,786	106.1	99.45	36.2	92.37	91.76
C187T	711,966,030	106.5	97.48	36.3	92.37	91.49
C194	685,901,760	102.7	99.45	34.9	91.71	91.31
C194T	630,151,208	94.3	99.45	32.1	91.71	91.17
C273	700,785,280	104.8	99.44	35.7	91.71	91.31
C273T	723,274,224	108.2	99.48	36.9	91.70	91.19
C288	759,582,872	112.5	99.05	46.1	91.72	87.21
C288T	739,642,796	109.7	98.98	43.7	91.31	87.08
C289	806,401,886	119.9	98.92	46.3	91.86	88.23
C289T	783,661,204	116.9	99.17	44.5	91.78	87.75
C330	989,225,366	148.0	99.39	49.3	92.17	90.73
C330T	795,117,586	118.9	99.41	39.6	92.03	88.68
C373	804,368,146	120.3	99.47	41.0	91.71	91.39
C373T	718,627,072	107.5	99.49	36.6	91.70	91.24
C379	792,789,536	117.8	99.04	45.8	91.24	89.67
C379T	785,647,292	117.2	99.17	44.8	91.21	88.80
C388	845,569,510	126.6	99.46	42.2	91.48	90.76
C388T	894,340,280	133.9	99.46	44.6	91.49	90.89
C396	788,264,984	116.9	99.07	46.4	91.83	87.85
C396T	716,228,232	106.7	99.19	42.5	91.71	86.09
C398	875,233,268	130.9	99.44	43.6	91.48	90.85
C398T	812,832,240	121.6	99.42	40.5	91.46	90.33
C404	745,870,020	111.7	99.41	37.9	92.37	91.86
C404T	833,925,190	124.9	99.45	42.4	92.37	91.88
C414	738,451,420	110.6	99.48	37.6	91.72	91.37
C414T	672,094,060	100.6	99.51	34.3	91.71	91.04
C418	755,099,972	111.6	98.85	45.5	91.54	87.05
C418T	756,706,394	112.8	99.10	43.9	91.18	86.69
C420	782,936,620	116.2	98.65	45.7	91.76	87.60
C420T	810,245,112	120.8	99.11	46.4	91.82	88.16
C429	564,994,088	85.30	99.78	30.0	96.40	86.42
C429T	811,508,188	122.5	99.80	42.9	94.90	88.92
C434	754,966,046	113.9	99.78	39.9	95.30	89.46
C434T	705,976,760	106.6	99.80	37.3	94.70	87.54
C449	804,269,940	120.5	99.42	41.0	92.38	92.01
C449T	687,106,384	102.9	99.47	35.0	92.38	91.54
C450	695,426,460	105.0	99.65	36.7	95.90	91.25
C450T	637,944,982	96.30	99.74	33.7	94.80	90.35
C459	686,371,123	102.8	99.20	35.0	91.71	91.27
C459T	702,077,558	105.2	99.39	35.8	92.36	91.44
C467	740,961,324	110.9	99.40	37.0	91.42	89.92
C467T	811,099,808	121.3	99.45	40.4	91.38	88.78
C469	786,984,946	116.6	98.83	45.9	91.78	87.75
C469T	775,160,750	114.9	98.92	46.1	91.17	87.87
C474	704,033,636	107.7	99.45	35.9	91.71	91.32
C474T	727,547,646	108.8	99.33	37.1	91.71	91.29
C476	706,761,594	105.8	99.44	35.3	91.88	86.89
C476T	781,669,314	117.0	99.45	39.0	91.87	88.84
C482	781,568,292	115.8	99.00	46.4	91.29	89.63
C482T	750,367,280	111.3	99.09	44.7	91.03	85.40
C484	636,624,780	94.60	98.94	37.1	91.11	85.60
C484T	778,587,768	115.6	99.10	46.4	91.25	88.51
C488	738,062,406	111.4	99.74	38.9	95.00	89.74
C488T	765,646,036	115.6	99.83	40.4	96.30	88.47
C497	827,634,716	122.8	99.04	48.0	91.33	90.11
C497T	772,559,334	115.2	99.14	44.2	91.22	89.44
C498	732,708,926	108.7	99.12	43.9	91.70	86.79
C498T	782,624,798	115.9	99.11	46.5	91.74	86.82
C500	811,693,756	120.7	99.10	46.3	91.89	88.28
C500T	739,230,362	109.7	98.89	43.8	91.17	87.87
C501	799,575,558	118.8	99.02	46.8	91.84	88.06
C501T	765,086,906	113.7	99.08	44.8	91.63	86.44
C506	754,784,530	111.8	98.82	45.3	91.80	87.29
C506T	720,805,784	107.4	99.00	42.4	91.25	85.75
C507	717,076,738	106.3	98.93	43.7	91.68	86.25
C507T	816,287,174	121.7	98.93	46.0	91.94	88.49
C511	740,833,912	111.8	99.78	39.1	96.00	87.93
C511T	715,283,644	108.0	99.75	37.8	94.90	87.26
C547	721,065,024	108.8	99.76	38.1	95.30	88.45
C547T	734,693,272	110.9	99.69	38.8	95.20	90.57
C554	600,706,148	87.31	99.32	32.0	91.06	84.49
C554T	600,706,148	84.65	99.46	32.0	90.49	83.00
C569	797,851,966	120.4	99.81	42.1	95.40	86.12
C569T	718,985,370	108.6	99.73	37.9	95.00	88.93
C570	655,831,072	99.00	99.67	34.6	94.40	90.57
C570T	830,993,866	125.5	99.74	43.9	95.20	91.54
C584	785,950,762	118.7	99.75	41.5	95.0	90.78
C584T	647,889,374	97.80	99.83	34.2	96.40	90.47
C594	735,103,200	111.0	99.34	38.8	94.00	91.65
C594T	554,483,974	83.70	99.78	30.0	96.50	89.65
C602	772,684,642	116.6	99.75	40.8	96.30	88.01
C602T	735,103,200	111.0	99.34	38.8	95.00	89.67
C649	683,345,166	103.1	99.82	35.9	91.33	84.35
C649T	727,873,090	109.9	99.83	38.2	93.16	84.69
C658	566,594,228	85.50	99.79	30.0	96.50	90.54
C658T	567,242,242	85.60	99.69	30.0	96.40	91.65
C662	635,677,938	95.90	99.71	33.6	94.00	87.46
C662T	760,943,510	114.9	99.82	40.2	96.50	87.01
C663	684,404,180	103.3	99.75	35.9	91.42	88.77
C663T	752,020,380	113.5	99.82	39.5	93.43	90.45
C666	673,308,798	101.6	99.82	35.3	92.24	86.35
C666T	659,271,254	99.50	99.70	34.6	93.00	87.46
C668	730,885,192	110.3	99.81	38.4	96.60	89.65
C668T	730,885,192	110.3	99.81	38.4	93.11	84.65
C669	538,920,270	81.40	99.83	30.0	96.40	89.53
C669T	606,534,910	91.60	99.85	32.0	96.80	88.75
C678	609,257,610	91.90	99.84	32.2	96.70	87.15
C678T	591,333,566	89.30	99.84	31.2	96.60	89.63
C679	826,097,192	124.7	99.82	43.3	91.67	89.55
C679T	716,931,988	108.2	99.68	37.6	96.41	90.45

T = tumor tissue, without T = corresponding blood DNA, or adjacent normal tissues.

### 3.3 Mutation rate and microsatellite status

**FIGURE 1:**
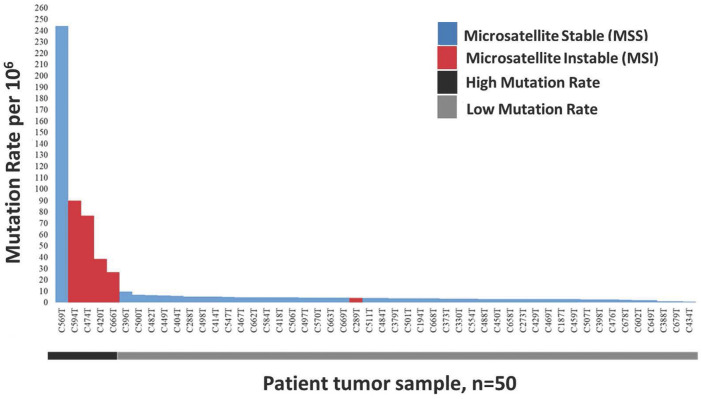
Distribution of 50 Microsatellite Instable (MSI) and Microsatellite Stable (MSS) CRCs sorted by decreasing mutation rate. Blue represents non MSI-High tumours consist of MSI-Low and MSS, while red represent MSI-High tumours.

In this study, we defined a high mutation rate or hypermutated as >12 mutations/Mb, in concordant as reported in TCGA 2012. The somatic mutation rates varied among the samples. The average mutation rate was 13.11 per Mb, with a range of 0.83–243.97 mutations per Mb. Of the 50 patients tested for microsatellite instability, 10% (*n* = 5) were classified as microsatellite instability-high (MSI-H), 10% (*n* = 5) as microsatellite instability low (MSI-L) and the remaining 80% (*n* = 40) were microsatellite stable (MSS). The median mutation rate in MSI-H, MSI-L and MSS groups were 57.7/Mb, 4.1/Mb and 3.8/Mb, respectively. The median mutation rate was significantly higher in MSI-H patients (*p* < 0.05) compared to MSI-L. Nearly all (4/5, 80%) MSI-H patients, except one (C289T), were classified as hypermutated and the association of MSI-H status with a high mutation rate was statistically significant (*p* < 0.001). Besides, one of the microsatellite stable (MSS) tumours, patient C569T, was hypermutated with a mutation rate of 243.97/Mb (Figure 1).

### 3.4 Somatic mutations landscape in malaysian colorectal cancer

**FIGURE 2:**
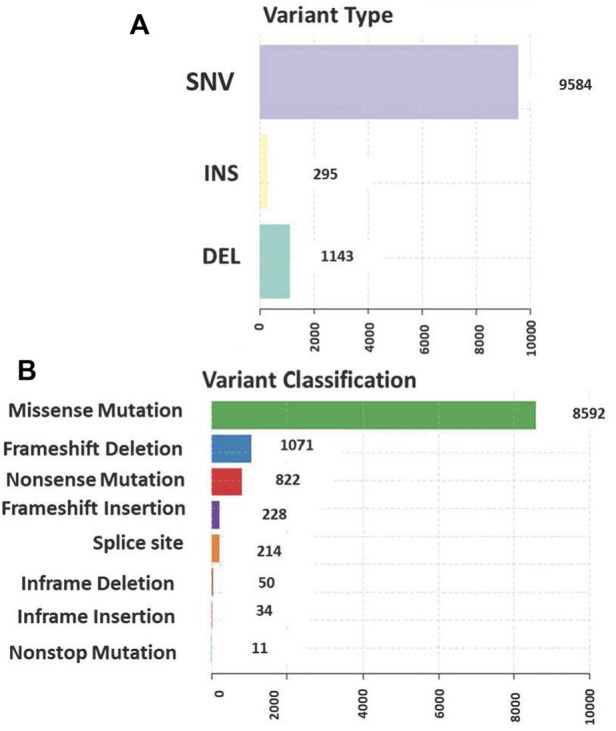
**(A)** Single nucleotide variants (SNVs) was the most common variant type detected **(B)** The distribution of the variants in genomic regions and types of exonic variants detected with missense mutation being the highest among all classes.

**FIGURE 3:**
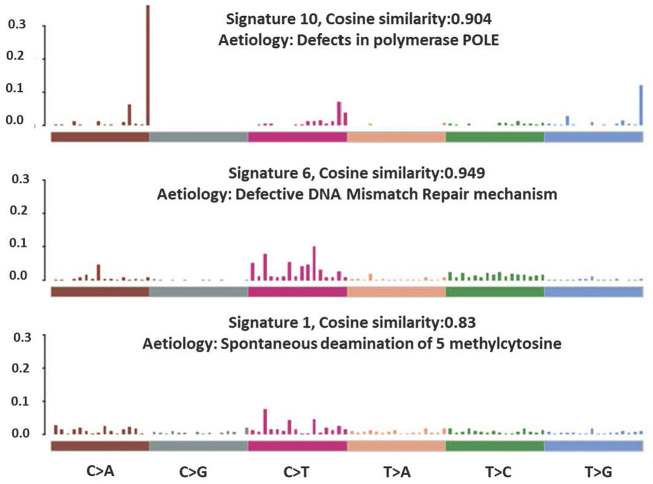
Mutational signatures identified in the CRC patients.

The total somatic variants detected in each CRC patient ranged between 2,587 and 756,750. Single nucleotide variants (SNVs) (Figure 2A) was the most variant type detected, with missense mutations being the highest variant class (Figure 2B). We analyzed the mutational signature underlying the development of our local CRC patients and three signatures similar to the COSMIC signature 6, 10 and 1 with cosine similarity of 0.949, 0.904 and 0.83, respectively, were discovered (Figure 3). The COSMIC signature six is related to defective DNA mismatch. COSMIC signature 10 is associated with defects in polymerase POLE, while signature one pertains to spontaneous deamination of 5-methylcytosine.

**FIGURE 4:**
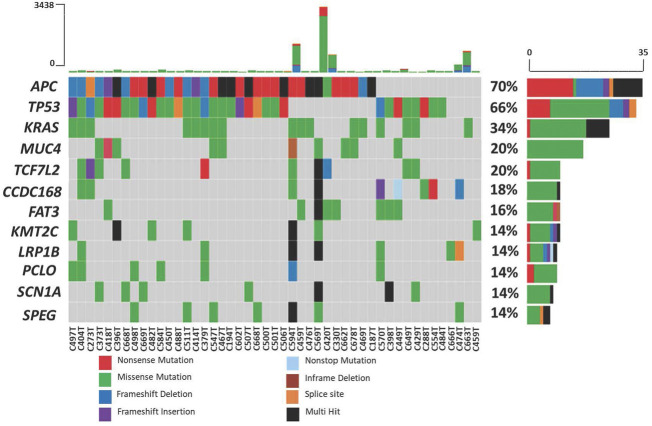
Mutation frequency in 50 Malaysian colorectal cancers. Each color in the boxes represent the mutation types.

The top ten most frequently mutated genes are APC, TP53, KRAS, MUC4, TCF7L2, CCDC168, FAT3, KMT2C, LRP1B, PCLO, SCN1A and SPEG. Mutations in three well-established CRC genes, APC, TP53 and KRAS, were present in 70%, 66% and 34% of the patients, respectively (Figure 4). Using MutSigCV, we identified significantly mutated genes and from this analysis, the number of significantly mutated genes with *p*-values less than 0.001, 0.01, and 0.05 were 15, 25, and 113, respectively. Among these, the top significantly mutated genes (*p* < 0.001 and q < 1.0) are APC, TP53, KRAS, TCF7L2 and ACVR2A. These genes, APC, KRAS and TP53, were mutated in more than 30% of the patients. The remaining two were mutated in less than 20%, namely TCF7L2 (20%, *p* < 0.0001, q = 0.02) and ACVR2A (8%, *p* < 0.0001, q = 0.63). The significantly mutated genes (SMGs) identified (*p* < 0.01 and q < 1.0) are summarized in Table 3.

**TABLE 3:** List of five top significantly mutated genes with *p* < 0.001 and q < 1.0.

Genes	*p*-value	q value	Frequency (%)	Samples
APC	0.00	0.00	75	C273T, C379T, C404T, C414T, C467T, C497T, C194T, C373T, C396T, C418T, C482T, C498T, C500T, C501T, C506T, C507T, C469T, C476T, C187T, C330T, C420T
TP53	0.00	0.00	71	C273T, C379T, C404T, C414T, C467T, C497T, C194T, C373T, C396T, C418T, C482T, C498T, C500T, C501T, C506T, C507T
KRAS	0.00	0.00	29	C273T, C379T, C404T, C414T, C467T, C497T, C469T, C476T
TCF7L2	0.00	0.02	20	C404T, C273T, C373T, C668T, C379T, C594T, C569T, C420T, C649T, C429T
ACVR2A	0.00	0.63	8	C420T, C474T, C594T, C666T

Upon variants prioritization, 64 were identified as recurrent in 54 genes of two to six patients. As expected, most of the recurrent variants were presented in well-established CRC genes such as KRAS, APC and TP53, for which nearly all (92%) are known variants reported in the dbSNP or COSMIC database. In 12% of the patients (6/50 patients), KRAS G12D was observed to be the most frequent variant, followed by ACVR2A K435fs (8%, 4/50 patients) and TP53 R175H (8%, 4/50 patients).

Eleven clinically significant variants, classified as pathogenic, were identified in five genes, which were *KRAS* (rs121913529, rs112445441, rs121913529), *APC* (rs587781392, rs587782518, rs121913332), *TP53* (rs28934576, rs121912651), *PIK3CA* (rs104886003) and *BRAF* (rs113488022). All of the mentioned variants are listed in Table 4. We identified 20 candidate driver genes using the oncodrive function in maftool v2.0.16. However, only two genes were significantly mutated (FDR<0.1), which were KRAS (G12D) and ACVR2A (K435fs). Remarkably, even with less than 20% of frequency, the ACVR2A gene was discovered to be one of the driver genes and was significantly mutated among other genes, suggesting its possible role in tumorigenesis of CRC in our local patients. The list of identified cancer driver genes is shown in Table 5.

**TABLE 4:** List of identified known and novel recurrent somatic variants with their clinical significance in CRC genomes.

Gene/Chr	Start	End	Ref	Alt	Samples	Amino acid change	COSMIC ID	dbSNP ID	Clinical significance
KDM4E/11	94,759,020	94,759,020	G	A	C434T	R100H	NA	NA	NA
					C569T				
KRAS/12	25,398,284	25,398,284	C	G	C414T	G12A	COSM1140134	rs121913529	Pathogenic
					C678T		COSM522		
TP53/17	7,577,120	7,577,120	C	T	C547T	R141H	COSM99729	rs28934576	Pathogenic
					C668T	R273H	COSM1645335		
							COSM3356963		
							COSM10660		
MUC16/19	9,015,323	9,015,323	C	A	C187T	L12755F	NA	NA	NA
					C330T				
MUC16/19	9,015,324	9,015,324	A	G	C187T	L12755S	NA	NA	NA
					C330T				
POTED/21	14,983,063	14,983,063	G	C	C662T	E172Q	NA	NA	NA
					C666T				
PIK3CA/3	178,936,091	178,936,091	G	A	C396T	E545K	COSM763	rs104886003	Pathogenic
					C398T		COSM125370		
APC/5	112,116,592	112,116,592	C	T	C187T	R223X	COSM13134	rs587781392	Pathogenic
					C506T	R213X			
APC/5	112,175,507	112,175,507	C	T	C467T	Q1406X	COSM19087	rs587782518	Pathogenic
					C501T				
APC/5	112,175,639	112,175,639	C	T	C569T	R1450X	COSM13127	rs121913332	Pathogenic
					C594T				
KRAS/12	25,398,281	25,398,281	C	T	C459T	G13D	COSM1140132	rs112445441	Pathogenic
					C467T		COSM532		
					C497T				
TP53/17	7,577,539	7,577,539	G	A	C414T	R116W	COSM3388183	rs121912651	Pathogenic
					C450T	R248W	COSM120007		
					C511T	R155W	COSM120006		
							COSM10656		
							COSM120005		
							COSM1640831		
BRAF/7	140,453,136	140,453,136	A	T	C396T	V28E	COSM476	rs113488022	Pathogenic
					C449T	V600E			
					C474T				
TP53/17	7,578,406	7,578,406	C	T	C404T	R43H	COSM3355994	rs28934578	Pathogenic
					C484T	R175H	COSM1640851		
					C501T	R82H	COSM99024		
					C649T		COSM99023		
							COSM10648		
							COSM99914		
							COSM99022		
ACVR2A/2	148,683,686	148,683,686	TA	T	C420T	K435fs	COSM252949	rs764719749	NA
					C474T				
					C594T				
					C666T				
KRAS/12	25,398,284	25,398,284	C	T	C273T	G12D	COSM521	rs121913529	Pathogenic
					C469T		COSM1135366		
					C547T				
					C570T				
					C649T				
					C663T				

**TABLE 5:** List of cancer driver genes analyzed using oncodrive function in maftools. FDR<0.1 indicates the most significantly mutated driver genes.

Gene	Total number of variants	Frequency (%)	*p* Value	FDR
KRAS	17	34	0.00	0.00
ACVR2A	5	10	0.00	0.00
TP53	33	66	0.04	0.29
CNTLN	5	6	0.18	0.35
DSCAM	5	10	0.18	0.35
FBXW7	5	10	0.18	0.35
IGSF3	5	8	0.18	0.35
JARID2	5	10	0.18	0.35
OCA2	5	10	0.18	0.35
PTPRS	5	10	0.18	0.35
PIK3CA	7	14	0.20	0.37
COL6A3	7	14	0.48	0.64
CSMD1	7	8	0.48	0.64
IGFN1	7	12	0.48	0.64
OTOGL	7	6	0.48	0.64
TNRC18	8	10	0.59	0.74
APC	49	70	0.69	0.81
SCN1A	10	14	0.73	0.81
MUC16	22	20	0.77	0.81
MUC4	14	20	0.85	0.85

### 3.5 Distribution of KDM4E, MUC16 and POTED hotspot and novel mutations

**FIGURE 5:**
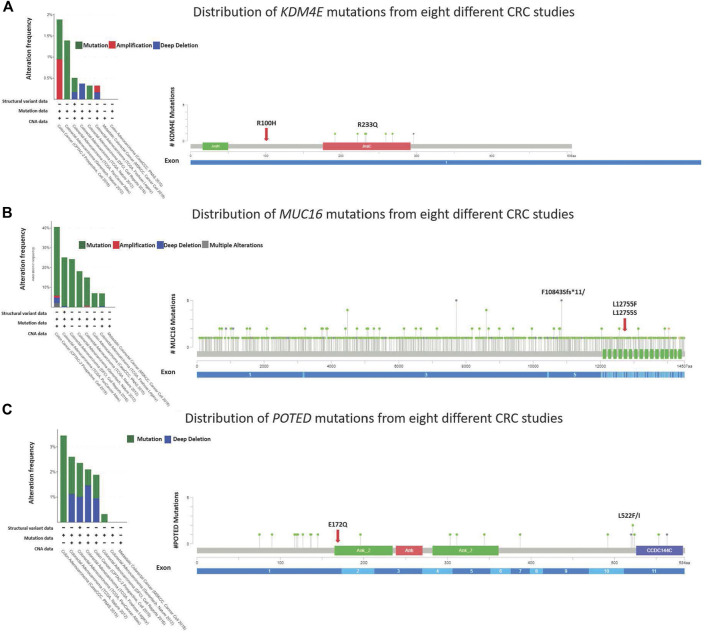
Lollipop diagram corresponding colorectal cancer studies selected in cBioportal and the frequency and types of changes occur.

Four novel, non-synonymous variants, were identified in three genes; KDM4E R100H, MUC16 L12755F and L12755S, and POTED E172Q. At the time of analysis, these variants had not been previously reported in neither COSMIC or dbSNP. The mutation hotspots of the genes were analyzed using the cBioPortal web-based tool (https://www.cbioportal.org/) (Cerami et al., 2012; Gao et al., 2013). The lollipop plot in Figures 5A–C shows the distribution and classes of hotspot mutations in these three genes across eight different CRC datasets (*n* = 5,323) (assessed on 30.08.2022). The red arrow indicates the location of the identified novel variant across protein domains of the genes.

### 3.6 Druggable somatic alterations

Based on the clinical annotation using *Cancer* Genome Interpreter, 88% (44/50) of the patients harboured at least one (range from 1 to 16) predicted candidate of druggable alterations. These alterations were either the targets of existing therapies (FDA guidelines or NCCN guidelines) or are currently being investigated in clinical trials (case reports, early trials, late trials and pre-clinical). Among them were various APC variants, detected in 72% (36/50) of the patients and predicted to respond against tankyrase inhibitors at the pre-clinical level. KRAS G12D was detected in 12% (6/50) of the patients and these patients were predicted to be resistant to several EGFR monoclonal antibody inhibitors (Panitumumab and Cetuximab) and ERBB2 monoclonal antibody inhibitor (Trastuzumab and Lapatinib). Six other KRAS variants were also observed in 10 (20%) different patients, which were predicted to be responsive to the combination of monoclonal antibody inhibitors such as MEK and PIK3 pathway inhibitors and MEK and MEK BCL-XL inhibitors.

Moreover, 14% (7/50) of CRC patients whose tumours possess PIK3CA variants were predicted to respond to the PI3K pathway inhibitor. However, these patients may not benefit from cetuximab therapy due to these variants. Four patients with different variants in the POLE gene might be suitable candidates for immunotherapy using the immune checkpoint inhibitor, PD1 antibody inhibitor. In addition, two *RNF43* mutations were discovered in one of the hypermutated phenotype patients, C474T. This patient is likely to be responsive to the Wnt pathway inhibitor, also known as the porcupine inhibitor.

### 3.7 RNF43 G156Afs mutation promotes colorectal cancer cells proliferation

**FIGURE 6:**
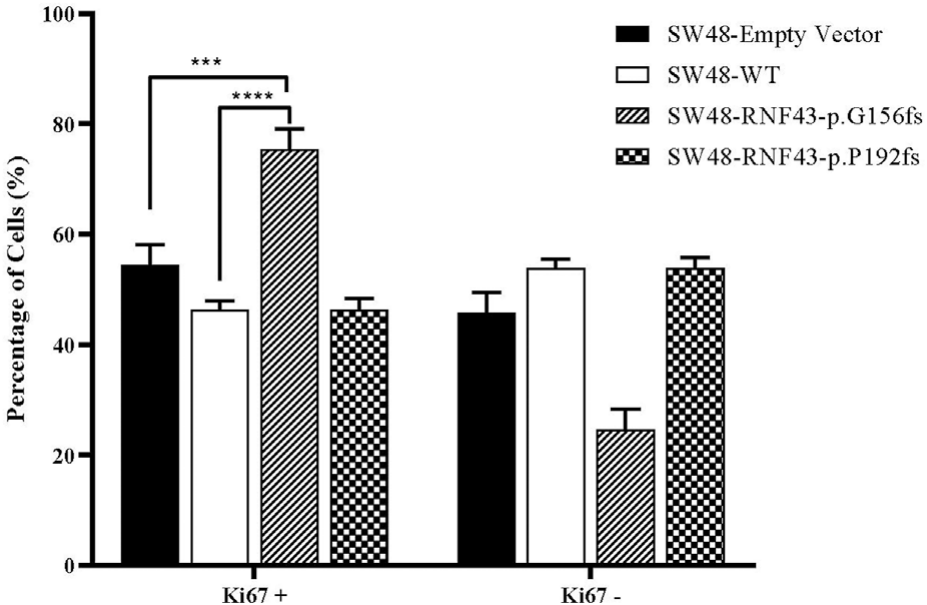
Percentage of proliferated cells assessed by Ki67 proliferation assay, 48 h post cell seeding. Percentage of Ki67+ was significantly higher in SW-RNF43-p.G156fs cells as compared to both SW48empty vector and SW48 wild type. Two-way ANOVA with Tukey′s range test, mean + SEM, *n* = 2, ***p < 0.005, ****p < 0.0001).

One commonly used marker for active cell proliferation is the Ki67 protein. To test the effect of harbouring the G156Afs and P192Gfs mutations on CRC cell’s proliferative capacity, we performed Ki67 FACS on the RNF43 wild type- and mutants-transduced SW48 cells. This was to compare the percentage of non-proliferating (Ki67^-^) and proliferating cells (Ki67^+^). We found that the expression of truncated RNF43 G156Afs promoted SW48 cells proliferation (78% of Ki67^+^cells) as compared to the SW48 cells transduced with empty vector (51.67% of Ki67^+^cells and wild-type RNF43 (57.4% of Ki67^+^cells). However, we did not observe any significant change in SW48 proliferative capacity between the cells expressing wild-type RNF43 and RNF43p.P192fs mutation (Figure 6).

### 3.8 RNF43 G156Afs and P192Gfs mutation increase sensitivity against LGK974 treatment

**FIGURE 7:**
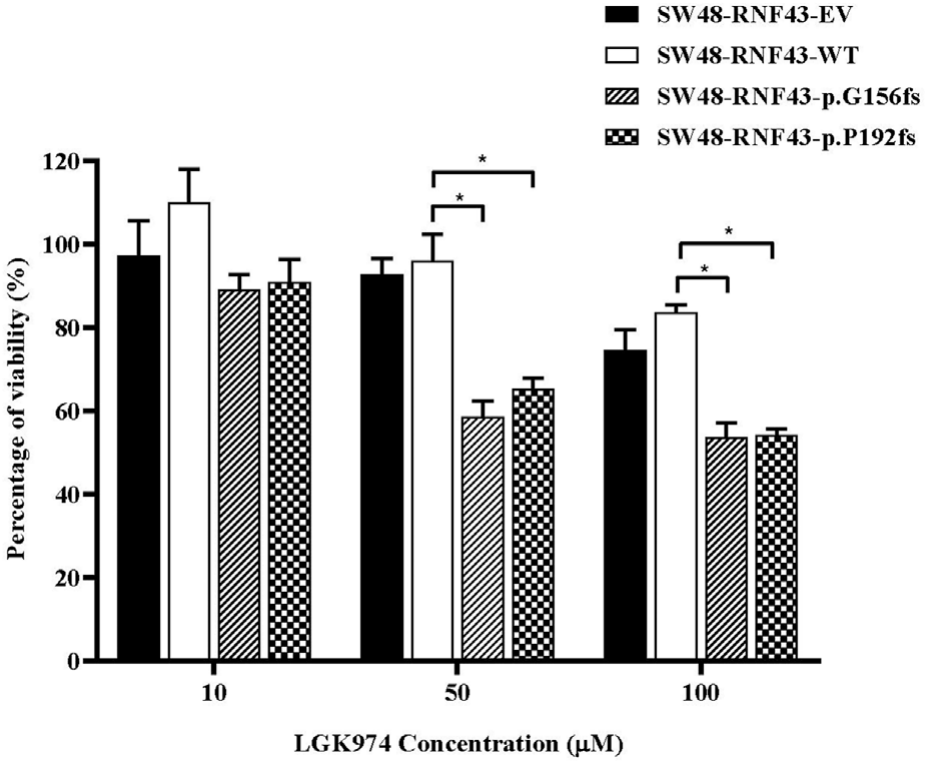
Mutants RNF43 promote reduction in cell viability. Statistical significance in all cases was measured by Two-way ANOVA with Tukey′s range test, (*p < 0.05), *n* = 3. Error bars represent average ± SD.

Several studies have shown the cells that carry the inactivating *RNF43* mutations are more sensitive to the porcine inhibitor LGK974 (Jiang et al., 2013; Tu et al., 2019; Zhong et al., 2019). Based on these reports, we were prompted to assess whether the expression of this *RNF43* G156Afs and P192Gfs mutations would sensitize the SW48 cells to LGK974 treatment. To this end, we performed a cell viability assay upon treating the SW48 cells that expressed empty vector, wild-type RNF43 and the two RNF43 mutations with increasing concentration of LGK974 drug (10–100 µM). We found that the cells that expressed these mutations were more sensitive to a higher concentration of LGK974 (50 μM and 100 µM) as compared to the cells that expressed empty vector and wild-type RNF43 (Figure 7). We, however, did not observe any significant difference or additive effect in terms of drug sensitivity between the 50µM and 100 µM LGK974 treatments. Therefore, we used the 50 µM LGK974 in the subsequent cell cycle arrest assay.

### 3.9 RNF43 G156Afs and P192Gfs mutation induce G1 cell cycle arrest upon LGK974 treatment

**FIGURE 8:**
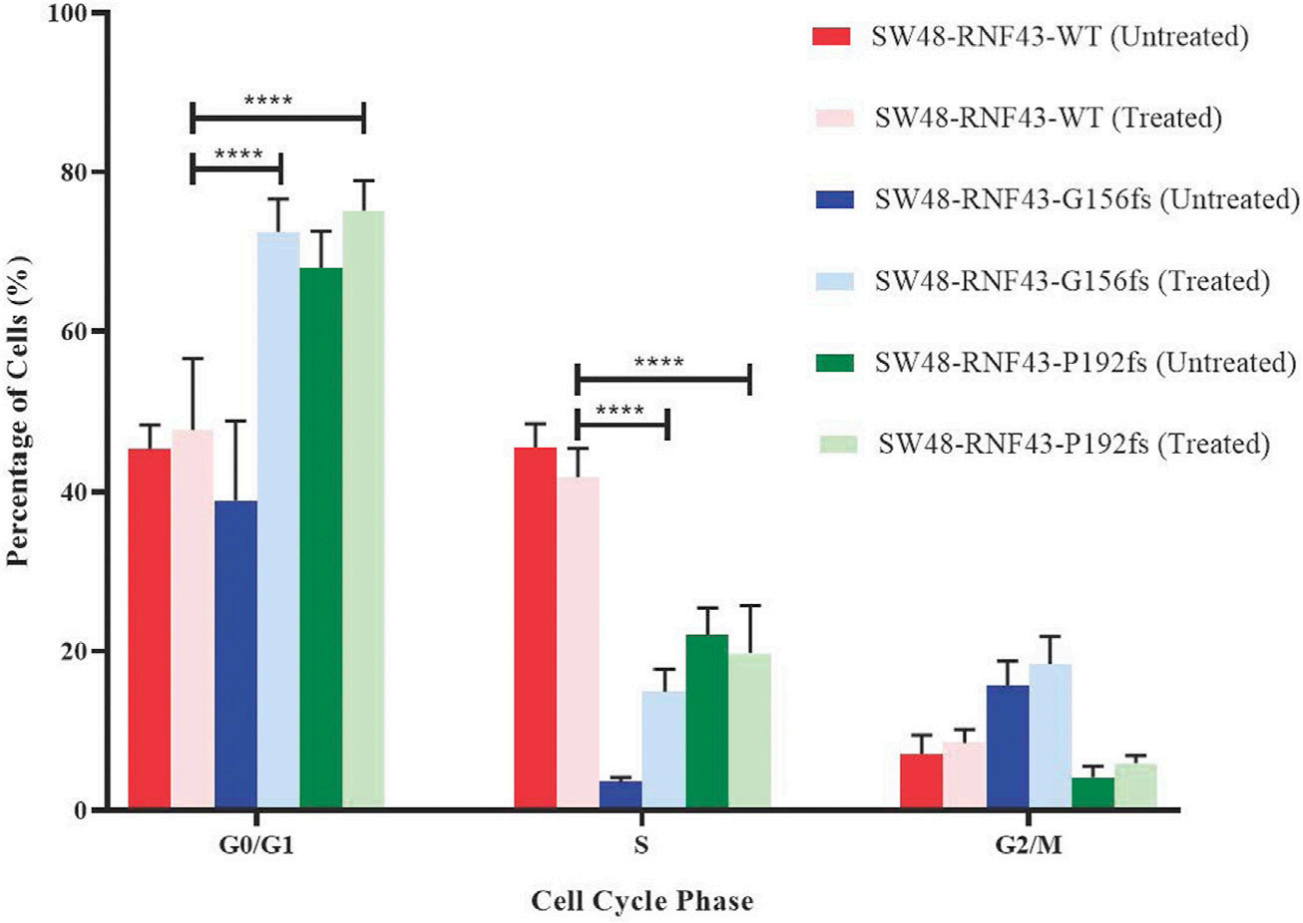
Distribution of SW48 transduced cells upon treatment with 50 μM of LGK974 throughout different cell cycle phase. Statistical significance in all cases was measured by mixed effect analysis with Tukey′s range test, (*p < 0.05), *n* = 4. Error bars represent average ± SD.

Since LGK974 is known to affect the cell cycle, we examined the effect of LGK974 treatment on the cell cycle process of each of these SW48 transduced cell lines. We treated these cells with 50 µM LGK974 for 48 h and assessed the cell cycle phases using FACS and BD Cycletest™ Plus DNA Reagent Kit (BD Biosciences, US). FACS analysis revealed a significant percentage of SW48-RNF43-p.G156fs and SW48-RNF43-p.P192s cells at the G0/G1 as compared to SW48-RNF43 wild-type cells, showing that the *RNF43* mutated cells were arrested at G0/G1 phase upon the treatment. Consequently, lower percentage of cells entered S and G2/M phase due to staying in G0/G1 phase (Figure 8).

## 4 Discussion

In this present study, we performed WGS on 50 paired tumour tissues and their corresponding blood DNA or adjacent normal tissues of Malaysian CRC patients. The comprehensive analysis of the WGS data, which consisted of SNVs and Indels, resulted in the discovery of recurrent and novel variants in Malaysian CRC patients. The somatic mutation rate varied between the CRC patients. However, nearly all patients with hypermutated tumours were microsatellite instable (MSI). In the TCGA study, more than half of the hypermutated tumours had high levels of MSI (MSI-H) due to somatic mutation in mismatch repair genes, MLH1 methylation or the CpG island methylation phenotype (CIMP) (The Cancer Genome Atlas Network, 2012). The determination of MSI status is essential, especially in metastatic CRC (mCRC), because of its prognostic and therapeutic implications. MSI status has also been considered as the biomarker for the immune checkpoint inhibitor treatment response (Nojadeh et al., 2018). Two of the FDA-approved immune checkpoint inhibitors for programmed cell death-1 protein (PD-1), pembrolizumab and nivolumab, had survival benefits in patients with mCRC and MSI-H (Le et al., 2015; Overman et al., 2017). Therefore, we postulated that our C420T patient, who has a high level of MSI and mutation in the DNA polymerase epsilon (POLE) gene (R573W), might be benefited from the immune checkpoint inhibitor therapy. A recent study reported a favourable clinical response to pembrolizumab in CRC patients who have metastatic disease and are intractable to FOLFOX and FOLFIRI treatments. These patients were characterized by MSS phenotype and *POLE* mutation, which highlighted the importance of genomic profiling and the determination of microsatellite status for an effective therapeutic purpose (Gong et al., 2017). In addition, the *POLE* mutations can also serve as a prognostic marker. Patients carrying these mutations have a significantly better overall survival than those with wild type, regardless of their microsatellite status and tumour mutation burden. *POLE* mutations also predict a good response to the immune checkpoint inhibitor treatment. Based on this evidence, a clinical trial on toripalimab in patients with several solid tumours, including CRC, with *POLE* mutations and non-MSI-H, has been initiated (NCT03810339) (Wang et al., 2019). We found that our C569T patients, who has hypermutated tumour, MSS phenotype and *POLE* mutation, is likely to have a responsive effect against immune checkpoint inhibitor through our druggable alteration analysis.

Our genome data can be classified into three mutation signatures, signatures 1, 6 and 10, which were supported by several other studies on sporadic CRCs (Jia et al., 2014; Nagahashi et al., 2016; Tubbs and Nussenzweig, 2017). Signature one is strongly associated with an endogenous mutational process initiated by spontaneous deamination of 5-methylcytosine due to the ageing process (Tubbs and Nussenzweig, 2017). This is reflected in our patients’ age, of which 92% (*n* = 46) of the recruited CRC patients were above 50 years old with an average age of 64. Signature 6 and 10, on the other hand, are associated with defective MMR and defective exonuclease activity of *POLE,* respectively. We observed that 10% (*n* = 5) of the recruited patients were categorized as MSI-H, with all of them having at least one known somatic mutation in either MMR or POLE genes, which may lead to impaired MMR and exonuclease activity of *POLE*, respectively.

The top frequently mutated genes identified in our CRC patients cohort were APC, KRAS, TP53 and MUC4, which were also readily reported in multiple studies (The Cancer Genome Atlas Network, 2012; Abdul et al., 2017; Chang et al., 2019; Mohd Yunos et al., 2019). High mutation frequency was also observed in several other genes, including CCDC168, FAT3, KMT2C, LRP1B, PCLO, SCN1A and SPEG. Based on the MutSigCV analysis, we also identified TCF7L2 and ACVR2A among the significantly mutated genes in our CRC patients. However, these two genes were not categorized as the top ten frequently mutated genes. In MutSigCV, SMGs were defined as the genes that are mutated more often than expected by chance of given background mutation processes. Our analysis indicated that most of the top ten frequently mutated genes were not statistically significant when mutational heterogeneity was considered. Despite their high mutation frequency in CRC, these genes may not be functionally important for tumorigenesis. Compared to other studies, the mutation frequency of *APC* and *TP53* in the Malaysian population was almost similar but much lesser than that of *KRAS* (The Cancer Genome Atlas Network, 2012; Abdul et al., 2017; Tanaka et al., 2017). Our previous genomic alterations profiling of Malaysian CRC patients also revealed that the APC gene was among the most frequently mutated gene, with a mutation frequency between 60% and 70% (Abdul et al., 2017; Chang et al., 2019).

On top of that, we identified four novel, non-synonymous which led to amino acid substitutions in three genes; KDM4E, MUC16 and POTED. Non synonymous variant of *KDM4E* R100H was identified in two patients C434T and C569T. KDM4 family protein functions as histone lysine demethylases that remove methyl groups from lysine residues in the histone tail, thereby controlling the transcriptional activity of target genes (Chen et al., 2006). KDM4 proteins family consist of four paralogues, namely *KDM4A*- *KDM4D*, and two pseudogenes, *KDM4E* and *KDM4F*. While *KDM4A* and *KDM4B* are the widely-studied members of the *KDM4* subfamily, the roles of *KDM4E* in cancers have rarely been reported (Wang et al., 2022a). Genomic alterations and overexpression of the *KDM4* family are reported in different breast cancer subtypes. Several *KDM4* inhibitors have already been used as anticancer drugs for breast cancers *in vitro* (Ye et al., 2015; Varghese et al., 2021). However, none of these drugs have undergone clinical trials yet (Varghese et al., 2021). The bioinformatics analysis demonstrated that the intronless *KDM4E* and *KDM4F* are expressed similarly to *KDM4D*. Because of their architecture and lack of expression, *KDM4E* and *KDM4F* are referred to as pseudogenes (Berry and Janknecht, 2013). Growing evidence that the pseudogenes have a variety of biological roles and that their dysregulation is frequently linked to human disorders like cancer signifies their potential as therapeutic targets (Prensner and Chinnaiyan, 2011; Wahlestedt, 2013; Sisu, 2021). Several genomic alterations of pseudogenes in CRC have been identified. For instance, pseudogenes *DUXAP8*, *MST O 2P* and *MYLKP1* involved in supporting CRC progression and enhance cancer risk (Lynn et al., 2018; He et al., 2020; Guo and Zhang, 2022). Hence, it is worth to explore the molecular characteristic and functional relevance of the identified recurrent *KDM4E* R100H mutation to unravel their potential as therapeutic target in CRC.

In this present study, we have identified a recurrent, non-synonymous *MUC16* L12755S mutation which was predicted to be deleterious by SIFT and PolyPhen-2 tools. Located within the tandem repeat domain, this particular mutation has not been previously reported in CRC and is worth exploring its functional relevance in future studies.The MUC16 gene encodes for a highly glycosylated protein that consists of two primary domains: a tandem repeat domain (interspersed with SEA domain) containing the CA-125 epitope and a transmembrane domain (Hattrup and Gendler, 2008; Felder et al., 2014). CA-125 is an FDA-approved serum biomarker used in monitoring cancer progression and treatment response, particularly in ovarian cancer (Bottoni and Scatena, 2015; Li et al., 2018; Charkhchi et al., 2020). MUC16 is the most frequently mutated gene in endometrial cancer (Hu and Sun, 2018), and its oncogenic properties have been investigated in several other cancers such as glioblastoma (Yang C. et al., 2019), gastric cancer (Huang et al., 2021) and colorectal cancer (Björkman et al., 2019). Meanwhile, knocking down *MUC16* in CRC cells impaired their growth and metastatic capability due to the deregulation of JAK2-STAT3 signalling pathway(Liu et al., 2022). Furthermore, a significant correlation between the *MUC16* mutation with tumour mutational burden and microsatellite status was shown in patients with gastric cancer (Zhang et al., 2022), colorectal cancer (Wang et al., 2020), and melanoma (Zhang et al., 2020; Wang et al., 2022b) which signifies the used of immune checkpoint inhibitor (ICI) in the treatment regimen.

To our knowledge, our study is the first to report novel *POTED* E172Q mutation in CRC, which were discovered in two of our patients. POTE family gene has at least ten paralogs, which encode for cancer testis antigens (CTAs) that are expressed in the germ cells of the adult testis, fetal ovary, prostate, placenta. Moreover, POTE gene family has been associated with the pathogenesis of various human cancers in which their expression is higher in cancer tissues as compared to normal tissues (Coulie et al., 2014; Sharma et al., 2019). Due to their low expression in normal tissues, POTEs are potential biomarker candidates for cancer progression and therapeutic targets (Redfield et al., 2013). *POTED,* also known as *ANKRD21*, is one of the paralogs of *POTE* located on chromosome 21. This gene is one of the 45 gene signatures for metastatic predictor in triple-negative breast cancer (TNBC) whereby the high expression of POTED was associated with poor prognosis (Kuo et al., 2012). Nevertheless, the mechanism regulating the POTED expression in cancer remains to be elucidated. In 2019, Shen et al. demonstrated an aberrant expression of *POTEE*, another paralog of POTE gene family, perturbed the SPHK1/p65 signalling axis that consequently promoted tumorigenesis by inhibiting apoptosis in CRC cells. Their study has highlighted the potential roles of *POTEE* as a novel biomarker for the diagnosis and intervention of CRC (Shen et al., 2019). Thus, the functional roles of other paralogs of POTE gene family, such as *POTED*, remain elusive and worth pursuing.

The Wnt signalling pathway is frequently activated in most CRC cases due to the loss of function mutations in the APC gene. *APC* mutations were discovered to be one of the potential biomarkers for sensitivity to tankyrase inhibitors in CRC. Tankyrase inhibitors enhance the degradation of *β*-catenin and inhibit cell proliferation in CRC cell lines that harbour APC mutations (Schatoff et al., 2019; Jang et al., 2020). In this study, we identified four previously reported pathogenic APC truncating mutations, namely the R223X, R213X, Q1406X and R1450X, which were predicted to be sensitive toward tankyrase inhibitors. We analyzed the druggability of the identified mutations, which were expected to be the target of either existing therapies or currently being investigated in clinical trials. The response of APC truncating mutations, such as Q1405X, in *in vivo* model was proven to be sensitive against tankyrase inhibitor, G007-LK, through WNT suppression due to tankyrase synthase inhibition (Schatoff et al., 2019). The finding demonstrates the importance of these *APC* mutations in CRC and an investigation into how these mutations can be translated for targeted molecular therapeutics is warranted.

Besides *APC*, we also identified two N-terminal truncating mutations in *RNF43*, specifically the G156Afs and P192Gfs. These variants were found in C474T patient who has wild-type *APC*, *KRAS* and *TP5*3, is hypermutated, and MSI-H phenotype. From our druggable alterations analysis, those with *RNF43* mutations were predicted to be responsive against the porcupine inhibitor LGK974. Even with a prevalence of less than 20% in CRC, *RNF43* has been described as one of the emerging predictive markers for treatment selection, especially in those with *BRAF* V600E mutations and MSI-H tumors with low *MLH1* expression (Jiang et al., 2013; Giannakis et al., 2014; Tu et al., 2019; Yunos et al., 2020). RNF43 gene has been functionally characterized in multiple cancers such as pancreatic (Jiang et al., 2013), gastric (Niu et al., 2015) and hepatocellular carcinoma (Xing et al., 2013). Depending on the type and position of the mutations in the gene, *RNF43* mutations can function as either positive or negative regulators of the Wnt/β-catenin signalling pathway (Li et al., 2020; Yu et al., 2020). The widely reported *RNF43* mutations in CRC are the R117fs and G659fs, which these mutations are commonly observed in serrated CRCs with mutated *BRAF* and MSI (Bond et al., 2016). The R117fs along with another RNF43 mutation, P441fs, act as positive regulators of the Wnt/β-catenin signalling pathway because the presence of these mutations resulted in FZD accumulation on the CRC cells surface. Furthermore, treatment with LGK974 decreased the Wnt/β-catenin activity induced by these mutations (Cho et al., 2022). Contrariwise, a study by Tu et al. showed that G659fs mutation does not confer any dominant-negative activities and is unlikely to play a role in supporting CRC pathogenesis (Tu et al., 2019). This finding was supported by several independent studies that show the same G659fs C-terminal truncation did not affect the Wnt/β-catenin signalling (Li et al., 2020; Cho et al., 2022). A more recent study has shed light on this observation whereby the G659fs actually promoted CRC cells growth *via* PI3K/mTOR instead of the Wnt signaling pathways (Fang et al., 2022). Collectively, these observations indicate that different RNF43 mutations would possess different molecular properties whereby each of these specific mutations warrant for comprehensive investigations in order to understand their roles in CRC.

Comprehensive screening of 135 *RNF43* missense and frameshift mutations in multiple human cancers revealed that all of the frameshift mutations and almost all missense mutations are located in the RING domain. This resulted in the RNF43 loss of function and subsequently increased activity of Wnt/β-catenin (Yu et al., 2020). Cho et al. demonstrated that even though RNF43 R117fs could interact with FZD5, RNF43 with this specific mutation could not ubiquitinate FZD5 due to the lack of the RING domain. It suggests that the RING domain of *RNF43* is vital for regulating the Wnt/β-catenin signalling pathway (Cho et al., 2022). Herein, we investigated the role of two *RNF43* mutations, G156Afs and P192Gfs, identified from our WGS data in the SW48 CRC cell line. The G156fs mutation is located in the protease-associated domain (PA domain) and mutation in this domain may affect the RING domain. Our observations revealed that *RNF43* G156Afs mutation, but not P192Gfs, promoted SW48 cell proliferation. Nevertheless, both mutations exhibited higher sensitivity to LGK974 treatment that was manifested *via* reduced cell viability and cell cycle arrest at the G0/G1 phase 48 h post-treatment. We have successfully characterized the potential roles of these truncating *RNF43* mutations in CRC pathogenesis, which can be further explored for the development of novel therapeutic targets in CRC. Moreover, it is essential to further validate invidual mutation identified from any genomic profiling studies to confirm their involvement in tumorigenesis. This is because Altogether, the analysis of druggable variants from our WGS, supported by the functional characterization, enhanced our understanding of the value of genomics and translating them into precision medicine.

## Data Availability

The data presented in the study are deposited in the NCBI SRA repository, accession number PRJNA928101.
